# Does Acute-on-Chronic Liver Failure Still Matter After Liver Transplantation? A Systematic Review and Meta-Analysis of One-Year Survival

**DOI:** 10.3390/diagnostics16142206

**Published:** 2026-07-15

**Authors:** Ethar Yousif, Jonathan Soldera

**Affiliations:** 1 MSc Program in Gastroenterology, University of South Wales in Association with Learna Ltd., Cardiff CF37 1DL, UK; yousifethar@gmail.com; 2 Department of Gastroenterology, Logan Hospital, Brisbane, QLD 4131, Australia

**Keywords:** acute-on-chronic liver failure, liver transplantation, one-year survival, cirrhosis, end-stage liver disease

## Abstract

**Background**: Acute-on-chronic liver failure (ACLF) carries high short-term mortality, and liver transplantation remains the only definitive treatment for selected patients. Whether ACLF continues to confer a survival disadvantage after transplantation remains clinically relevant for graft allocation, candidate selection, and prognostic counselling. **Methods**: This systematic review and meta-analysis evaluated one-year survival after liver transplantation in patients with ACLF compared with non-ACLF transplant recipients. PubMed was searched for eligible studies reporting post-transplant survival outcomes. Data were extracted on study design, population, ACLF definition, comparator group, and one-year survival. Pooled survival proportions were calculated separately for ACLF and non-ACLF groups using random-effects models. Comparative survival was assessed using pooled risk ratios. Heterogeneity and publication bias were evaluated using I^2^, τ^2^, funnel plot inspection, and Egger’s test. **Results**: Ten studies including 59,686 liver transplant recipients were included, of whom 25,016 had ACLF and 34,670 did not. The pooled one-year survival after liver transplantation in ACLF patients was 78.8% (95% CI: 70.1–85.4), with substantial heterogeneity (I^2^ = 93.1%). In non-ACLF recipients, pooled one-year survival was 86.9% (95% CI: 75.3–93.5), also with high heterogeneity (I^2^ = 97.8%). Direct comparison showed lower one-year survival in ACLF recipients than in non-ACLF recipients, with a pooled risk ratio of 0.93 (95% CI: 0.92–0.94; *p* < 0.0001). Egger’s test did not suggest significant publication bias. **Conclusions**: ACLF does still matter after liver transplantation. Transplanted ACLF patients achieve clinically meaningful one-year survival, supporting transplantation as a valid treatment in selected candidates, but their survival remains lower than that of non-ACLF recipients. The implication is not that ACLF should exclude transplantation, but that ACLF severity, organ failure burden, infection status, and perioperative risk must be integrated more explicitly into selection and allocation decisions.

## 1. Introduction

Acute-on-chronic liver failure (ACLF) is not simply advanced cirrhosis with a worse laboratory profile. It is a distinct syndrome in which an acute precipitating event occurs on the background of chronic liver disease and is followed by hepatic and extrahepatic organ failure. Its clinical importance lies in the speed of deterioration. Patients who may appear to have a reversible decompensation can progress rapidly to renal, cerebral, circulatory, respiratory, or coagulation failure, with short-term mortality driven more by systemic failure than by liver dysfunction alone. This is why ACLF remains one of the most difficult syndromes in hepatology: it is common, unstable, heterogeneous, and frequently time-critical.

The definition of ACLF is still not uniform. The Asian Pacific Association for the Study of the Liver defines ACLF mainly through acute hepatic insult, jaundice, coagulopathy, and the development of ascites or encephalopathy within a short interval in a patient with known or unknown chronic liver disease. The European Association for the Study of the Liver-Chronic Liver Failure Consortium definition is centred on acute decompensation of cirrhosis complicated by organ failure. The North American Consortium for the Study of End-Stage Liver Disease uses a more infection- and organ-failure-based framework. These differences are not semantic. They influence who is labelled as ACLF, how severity is graded, how transplant urgency is interpreted, and how outcomes are compared across studies. The EASL-CLIF approach, particularly through CLIF-SOFA and CLIF-C ACLF, has gained wide acceptance because it links the diagnosis to measurable organ failure and short-term prognosis [1,2,3].

This prognostic framing matters because ACLF is a mortality syndrome before it is a transplant indication. Several studies have shown that organ-failure scores predict outcomes better than conventional liver-only measures in many ACLF settings. CLIF-SOFA, CLIF-C ACLF, and CLIF-C AD have been associated with mortality across acute decompensation and ACLF cohorts, and CLIF-SOFA has often performed particularly well for short-term risk prediction [2]. Brazilian cohort data have also reinforced the same principle: in decompensated cirrhosis, spontaneous bacterial peritonitis, and variceal haemorrhage, the presence of ACLF and higher organ-failure scores identify patients with markedly worse survival [4,5,6,7,8,9]. More recent multicentre data further suggest that the available prognostic models retain only moderate discriminatory ability in hospitalized decompensated cirrhosis, which is relevant when these scores are used to guide high-stakes decisions such as transplant listing [10]. In short, ACLF is prognostically real, but its measurement remains imperfect.

The pathobiology explains part of this instability. ACLF usually follows an acute insult, but the precipitant is not always identified. Bacterial infection, alcoholic hepatitis, gastrointestinal bleeding, hepatitis B reactivation, drug-induced liver injury, ischaemic injury, and portal vein thrombosis are recognised triggers. In many patients, however, no single cause is found. Western cohorts are more often dominated by alcohol-related liver disease, whereas hepatitis B remains a major driver in parts of Asia. The common final pathway is systemic inflammation, immune dysfunction, circulatory disturbance, and organ failure. Patients with higher MELD scores and more advanced baseline cirrhosis are more vulnerable, but ACLF cannot be reduced to MELD alone. The inflammatory response may be triggered by infection, bacterial translocation, tissue injury, or sterile inflammatory pathways, and is often accompanied by raised leukocyte count, C-reactive protein, cytokine activation, oxidative stress, and mitochondrial dysfunction [11,12,13,14,15].

The clinical expression of ACLF is therefore broad, but renal dysfunction and infection dominate much of the practical risk. Acute kidney injury, hepatorenal syndrome, hepatic encephalopathy, circulatory failure, respiratory failure, and coagulopathy all alter transplant candidacy and perioperative risk. Hepatorenal syndrome remains one of the most severe complications of cirrhosis, and the use of structured diagnostic and therapeutic protocols has been associated with improved survival and lower use of terlipressin and albumin without an apparent mortality penalty [16,17,18]. Sepsis adds another layer of risk. Cirrhotic patients admitted to intensive care with sepsis have high mortality, and early source control, antibiotics, albumin-based resuscitation, vasopressors, and careful biomarker interpretation remain central to management [19]. These points are not peripheral to transplantation. They determine whether a patient with ACLF is too sick to transplant, not sick enough to prioritise, or within the narrow window in which transplant offers meaningful survival.

Liver transplantation is the only definitive treatment for selected patients with ACLF. Supportive care may reverse the precipitating event and stabilize organ failure, but it does not correct the underlying end-stage liver disease. This creates an uncomfortable clinical problem. Without transplantation, mortality in severe ACLF is high; with transplantation, perioperative risk is also high, donor organs are scarce, and futility must be avoided. Adult liver transplantation has achieved excellent general outcomes, with reported one- and five-year survival around 94% and 83%, respectively, but these figures cannot simply be applied to ACLF recipients, who often arrive to surgery with infection, renal replacement therapy, vasopressor exposure, mechanical ventilation, or multiple organ failures [20]. The question is not whether transplantation works in liver failure. It does. The question is whether ACLF continues to impose a survival disadvantage after transplantation, and whether that disadvantage is large enough to change selection and allocation decisions.

Existing data suggest that liver transplantation can rescue many patients with ACLF, including those with ACLF grade 2 or grade 3, but often at the cost of greater perioperative complexity. ACLF has been associated with longer intensive care stays, a higher infection burden, prolonged renal replacement therapy, biliary and vascular complications, acute cellular rejection, and greater resource use after transplantation.

Some reports describe post-transplant survival in selected ACLF cohorts as comparable with that of patients with less severe cirrhosis, whereas others show a measurable survival decrement compared with non-ACLF recipients [21,22]. This apparent discrepancy is likely explained by selection. Transplanted ACLF patients are not representative of all patients with ACLF; they are the subgroup who survived long enough to undergo assessment, were considered suitable for transplantation, and obtained access to a graft. Therefore, post-transplant outcomes may appear favourable while still remaining inferior to those of non-ACLF recipients.

This distinction is central to organ allocation. Prioritising ACLF patients may be life-saving because waitlist mortality is high and standard allocation scores may underestimate the risk associated with extrahepatic organ failure. However, priority cannot be based on urgency alone. Allocating a donor liver to a patient with irreversible multiorgan failure, uncontrolled sepsis, or prohibitive frailty may not translate into meaningful survival.

Models such as the transplant arterial lactate and mechanical ventilation score for ACLF-3 have attempted to define the limits of transplant benefit using variables including age, lactate, leukocyte count, and mechanical ventilation. Ongoing infection, severe circulatory failure, renal failure, and alcohol-related contraindications remain important practical barriers in many transplant systems, although controlled infection should not automatically exclude transplantation [23,24,25]. The clinical task is not to deny transplantation to patients with ACLF, but to identify the point at which urgency, reversibility, and expected post-transplant survival intersect.

The broader transplant context also matters. Liver transplantation is limited by donor availability, regional allocation systems, and centre-specific thresholds. MELD-based allocation improved objectivity but does not fully capture inflammatory burden, infection, mechanical ventilation, vasopressor requirement, or the trajectory of organ failure. Older allocation discussions focused on classical indications, surgical feasibility, graft type, immunosuppression, and general post-transplant complications. These remain relevant, but they are insufficient for ACLF. In ACLF, the relevant decision is dynamic and often occurs in intensive care. It includes whether the precipitant has been controlled, whether renal failure is reversible, whether simultaneous liver-kidney transplantation is justified, whether infection is treatable, and whether the patient has crossed from high-risk benefit into probable futility [26,27,28,29]. This is why one-year survival remains a useful endpoint. It is crude, but it is clinically meaningful. It captures whether the transplant achieved more than short-term rescue.

The available literature is clinically important but scattered. Individual studies differ in ACLF definition, transplant type, recipient severity, donor source, regional practice, and comparator group. Some include ACLF-1 to ACLF-3, while others focus on critically ill or urgent-listing populations. Some compare transplanted ACLF patients with non-transplanted ACLF patients, while others compare ACLF with non-ACLF transplant recipients. This heterogeneity limits simple interpretation. Nevertheless, the core question remains relevant for clinical practice and policy: after a patient with ACLF receives a liver transplant, does ACLF still matter for one-year survival, or does transplantation largely neutralise the pre-transplant risk?

This systematic review and meta-analysis was designed to answer that question. The primary aim was to evaluate one-year survival after liver transplantation in patients with ACLF and to compare it with one-year survival in non-ACLF liver transplant recipients. The purpose was not to restate that transplantation is beneficial in end-stage liver disease, which is already established. The purpose was to quantify whether ACLF remains associated with a post-transplant survival penalty despite transplantation, and to clarify what that means for candidate selection, prognostic counselling, and organ allocation.

## 2. Materials and Methods

This systematic review and meta-analysis was conducted according to the Preferred Reporting Items for Systematic Reviews and Meta-Analyses 2020 statement (Table S1) [30]. The review protocol was registered in PROSPERO before completion of the review: CRD420251113730. The review question was structured using the PICO framework.

### 2.1. Eligibility Criteria

Studies were eligible if they included adult patients aged 18 years or older with acute-on-chronic liver failure who underwent liver transplantation and reported post-transplant survival. The intervention of interest was liver transplantation, including deceased donor and living donor transplantation where applicable. The comparator was liver transplantation performed in patients without ACLF. The primary outcome was one-year post-transplant survival.

Eligible study designs included cohort studies, case–control studies, registry-based studies, and randomized studies if available. Studies were restricted to human studies published in English. Studies published before 2000 were excluded. Case reports, animal studies, conference abstracts without full-text data, narrative reviews, systematic reviews, editorials, and studies without extractable post-transplant survival data were excluded. Studies were also excluded when ACLF patients were not transplanted, when the comparator group was not relevant to the review question, or when the reported data were insufficient for calculation of survival proportions or comparative effect estimates.

### 2.2. Search Strategy and Study Selection

PubMed, Scielo and Cochrane Library were searched using Medical Subject Headings and free-text terms related to liver transplantation, ACLF, survival, prognosis, and transplant selection. The search strategy was:

(“Liver Transplantation”[MeSH] OR “Liver Transplant”[tiab] OR “Hepatic Transplant”[tiab]) AND (“Acute-on-Chronic Liver Failure”[MeSH] OR “ACLF”[tiab] OR “acute on chronic liver failure”[tiab]) AND (“Outcomes”[tiab] OR “Survival”[MeSH] OR “Morbidity”[MeSH] OR “Mortality”[MeSH] OR “Prognosis”[MeSH]) AND (“Patient Selection”[MeSH] OR “Candidate Selection”[tiab] OR “Transplant Criteria”[tiab] OR “End Stage Liver Disease”[MeSH] OR “MELD score”[tiab] OR “CLIF-C score”[tiab]).

Reference lists of relevant articles were also checked manually to identify additional studies. Retrieved records were screened by title and abstract. Full texts were then assessed for eligibility. Duplicates were removed before final inclusion. The study selection process was reported using a PRISMA flow diagram [30].

### 2.3. Data Extraction

Data were extracted into a structured table. The following variables were collected: first author, year of publication, country, study design, study setting, number of included patients, ACLF definition, ACLF grade where available, donor type, transplant type, comparator group, number of ACLF and non-ACLF transplant recipients, one-year survival in each group, and the number of patients achieving the outcome in each arm. Where available, data on pre-transplant severity, MELD score, organ failure burden, renal replacement therapy, infection, intensive care admission, and post-transplant complications were also extracted.

The extracted data were checked for consistency with the reported study outcomes. When studies reported mortality rather than survival, one-year survival was calculated as the complement of one-year mortality, provided that the denominator was clear. Studies were not included in the quantitative synthesis when the survival outcome could not be extracted or derived with sufficient confidence.

### 2.4. Outcomes

The primary outcome was one-year survival after liver transplantation in patients with ACLF. The main comparative outcome was one-year survival in ACLF liver transplant recipients versus non-ACLF liver transplant recipients. Secondary descriptive outcomes included reported post-transplant complications, infection, renal replacement therapy, intensive care burden, and other clinical outcomes when available. These secondary outcomes were not pooled quantitatively unless sufficient homogeneous data were available.

### 2.5. Risk of Bias and Quality Assessment

Risk of bias was assessed using the ROBINS-I tool for non-randomised studies of interventions. Seven domains were evaluated: confounding, selection of participants, classification of interventions, deviations from intended interventions, missing data, measurement of outcomes, and selection of reported results. Each domain and the overall judgement were classified as low, moderate, serious, or critical risk of bias. Results were summarised in a study-level table and visualised using robvis traffic-light and weighted summary plots.

### 2.6. Statistical Analysis

The meta-analysis assessed one-year post-liver transplantation survival in patients with and without ACLF. First, pooled survival proportions were calculated separately for ACLF and non-ACLF recipients. Second, comparative survival between ACLF and non-ACLF recipients was assessed using pooled risk ratios.

Analyses were performed in R (version 4.6.0) using the meta package. Pooled proportions were calculated using the inverse variance method. Proportions were logit-transformed to stabilise variances, and Clopper–Pearson 95% confidence intervals were calculated for individual studies. Random-effects models were used because clinical and methodological heterogeneity was expected across studies, particularly in ACLF definition, transplant selection, donor type, severity of illness, and regional allocation practice. Between-study variance was estimated using restricted maximum likelihood. Hartung–Knapp adjustment was applied to provide more conservative confidence intervals for pooled estimates.

Heterogeneity was assessed using τ^2^, I^2^, H, and the Q test. Forest plots were generated for pooled ACLF survival, pooled non-ACLF survival, and the comparative risk ratio. Publication bias and small-study effects were assessed by visual inspection of funnel plots and Egger’s regression test. A *p* value below 0.05 was considered statistically significant.

## 3. Results

**Figure 1:**
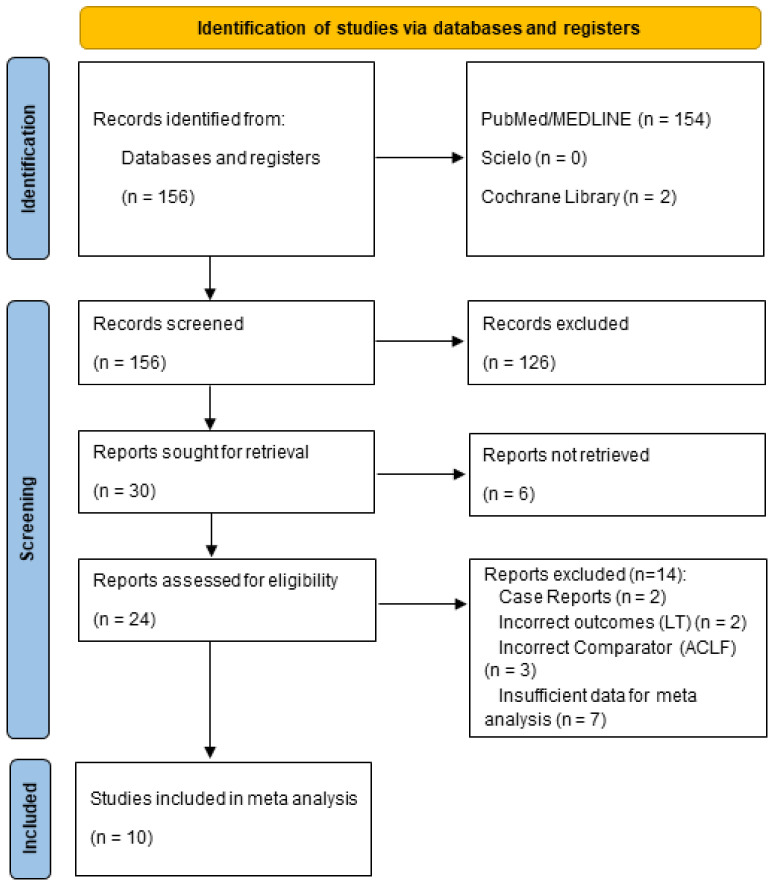
PRISMA 2020 flow diagram of study identification, screening, eligibility assessment, and inclusion.

The database search identified 156 records. After screening and eligibility assessment, 17 studies were included in the review and 10 in the meta-analysis (Figure 1). Thirty reports were selected for retrieval, six were not retrieved, and 24 full-text reports were assessed. Seven reports were excluded at full-text stage because they did not evaluate ACLF, did not include transplanted ACLF patients, were review-type publications, or did not report extractable one-year post-transplant survival data. The final quantitative dataset included 59,686 liver transplant recipients, of whom 25,016 had ACLF and 34,670 did not. The included studies were clinically heterogeneous, with variation in country, ACLF definition, transplant setting, donor type, disease severity, and comparator group (Table 1).

The included evidence came from a broad range of transplant settings. Studies were conducted in Brazil, Germany, China, Korea, the United States, Austria, Romania, and Vietnam, with a predominance of high-volume transplant centres and registry-based cohorts (Table 1). The sample size varied markedly between studies. Several cohorts were small or moderate in size, such as Cronst et al. [31], Goosmann et al. [32], Finkenstedt et al. [39], Iacob et al. [40], Bahirwani et al. [42], and Vu et al. [43], whereas larger datasets came from Asian transplant programmes and United States registry analyses, particularly Kwon et al. [45], Sundaram et al. [46], and Chok et al. [47]. This imbalance is important because the pooled results were influenced not only by survival probability, but also by the precision and weight of larger studies.

The clinical scenarios also differed. Some studies specifically evaluated post-transplant outcomes in ACLF recipients, while others assessed broader transplant populations and extracted ACLF subgroups. Several studies focused on living donor liver transplantation, including Moon et al. [35], Vu et al. [43], and Chok et al. [47], whereas others included deceased donor transplantation, urgent transplant evaluation, or mixed transplant pathways. Comparator data were not available for all studies. Some cohorts reported one-year survival only among ACLF recipients, while others provided direct ACLF versus non-ACLF comparisons. This explains why the proportional analyses and the comparative risk-ratio analysis were not identical in structure. It also reinforces that the pooled estimates should be read as aggregate transplant outcomes across heterogeneous clinical settings rather than as a single uniform ACLF population.

**Figure 2:**
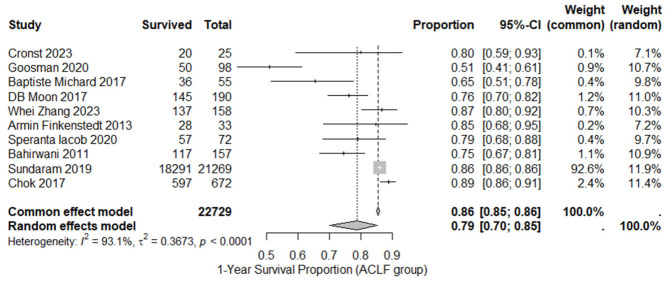
Forest plot of pooled one-year survival after liver transplantation in ACLF recipients [31,32,34,35,36,39,40,42,46,47].

Across the included studies, one-year survival after liver transplantation remained clinically meaningful in ACLF recipients, but it was lower than in non-ACLF recipients. In the pooled random-effects analysis, the one-year survival proportion among ACLF transplant recipients was 78.8% (95% CI: 70.1–85.4) (Figure 2). This estimate indicates that most transplanted ACLF patients survived to one year, despite the high-risk nature of the syndrome. However, the survival estimate was not homogeneous across cohorts. Between-study heterogeneity was substantial (I^2^ = 93.1%; τ^2^ = 0.3673), which was expected given the differences in ACLF grade, transplant urgency, regional allocation systems, donor source, and recipient severity. Individual study estimates ranged from approximately 51% in Goosmann et al. [32] to nearly 89% in Chok et al. [47], showing that post-transplant survival in ACLF is strongly shaped by selection and context.

The spread of individual study estimates is clinically informative. Studies from highly selected living donor transplant settings tended to report favourable post-transplant survival, while cohorts including urgent, critically ill, or higher-severity ACLF recipients showed lower survival. This pattern is consistent with the biology of ACLF and with transplant decision-making: the benefit of transplantation is substantial when organ failure is still reversible or controlled, but survival decreases as systemic illness, infection, renal replacement therapy, circulatory failure, or mechanical ventilation accumulate. Therefore, the pooled survival estimate of 78.8% should not be interpreted as a guarantee of transplant success in all ACLF patients. It reflects outcomes in patients who were selected, listed, transplanted, and followed within transplant-capable systems.

**Figure 3:**
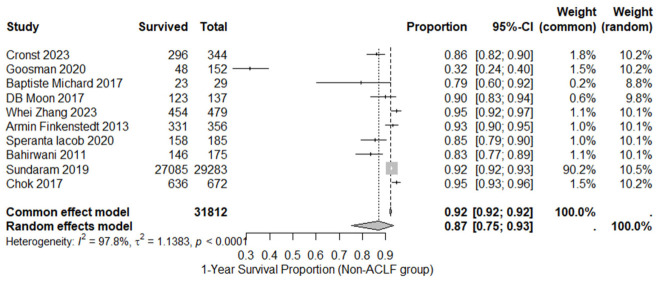
Forest plot of pooled one-year survival after liver transplantation in non-ACLF recipients [31,32,34,35,36,39,40,42,46,47].

In the non-ACLF transplant group, pooled one-year survival was higher, at 86.9% (95% CI: 75.3–93.5) (Figure 3). Heterogeneity was also high in this analysis (I^2^ = 97.8%; τ^2^ = 1.1383), again reflecting differences in study population, transplant indication, donor type, and follow-up structure. Several studies reported one-year survival above 90% in non-ACLF recipients, particularly in cohorts where the comparator group represented elective or less systemically unwell transplant candidates. This finding is expected. Non-ACLF recipients may have severe liver disease, but they do not carry the same burden of acute systemic organ failure at transplantation. The higher pooled survival in this group therefore provides a clinically plausible comparator rather than an unexpected result.

**Figure 4:**
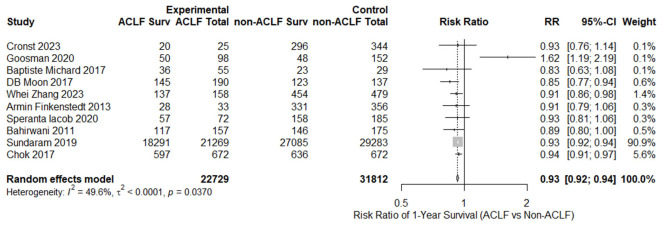
Forest plot comparing one-year survival after liver transplantation in ACLFversus non-ACLF recipients [31,32,34,35,36,39,40,42,46,47].

Direct comparison using pooled risk ratios confirmed that ACLF remained associated with lower one-year survival after liver transplantation. The pooled risk ratio for one-year survival in ACLF versus non-ACLF recipients was 0.9295 (95% CI: 0.9179–0.9413; *p* < 0.0001) (Figure 4). In practical terms, ACLF recipients were approximately 7% less likely to survive to one year after transplantation than non-ACLF recipients. This is not a large enough difference to argue against transplantation in selected ACLF patients, but it is large enough to matter for counselling, allocation, and perioperative risk stratification. The finding supports the central message of the review: ACLF does still matter after transplantation, but it does not make transplantation futile.

The comparative analysis also showed less heterogeneity than the separate pooled survival analyses. Heterogeneity for the risk-ratio analysis was moderate (I^2^ = 49.6%), suggesting that although absolute survival varied widely between cohorts, the direction of effect was more consistent when ACLF and non-ACLF recipients were compared within the same study environment. This is important because within-study comparisons partly control for centre practice, donor allocation, surgical pathway, follow-up, and regional healthcare factors. The consistency of the comparative result makes the survival penalty associated with ACLF more credible than a simple comparison of pooled survival proportions alone.

**Figure 5:**
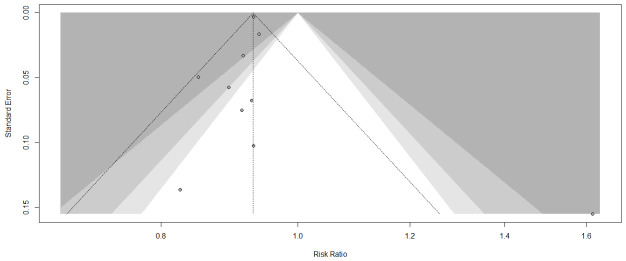
Funnel plot assessing small-study effects and publication bias for the comparative survival analysis.

Assessment of small-study effects did not suggest relevant publication bias. The funnel plot was broadly symmetrical, and Egger’s regression test was not significant (*p* = 0.9899) (Figure 5). These findings support the stability of the main comparative result. Still, they do not remove the main limitation of the evidence base, which is clinical heterogeneity. Most included studies were observational, several were retrospective, and ACLF definitions, transplant thresholds, and comparator groups were not uniform. The results therefore support a cautious but clinically useful conclusion: transplanted ACLF patients have acceptable one-year survival, but their outcomes remain inferior to those of non-ACLF transplant recipients.

**Figure 6:**
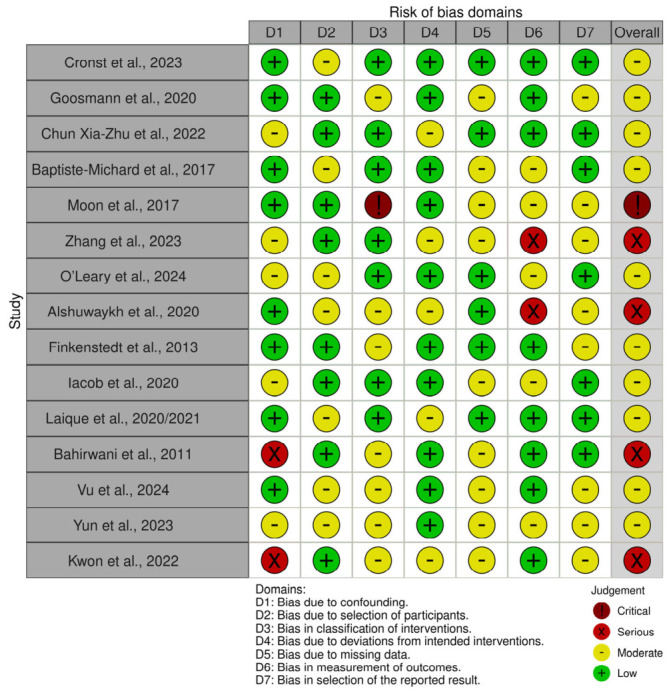
Risk-of-bias summary across included non-randomised studies assessed using ROBINS-I [31,32,33,34,35,36,37,38,39,40,41,42,43,44,45].

**Figure 7:**
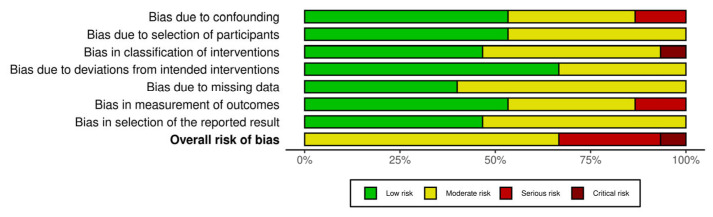
Study-level ROBINS-I risk-of-bias assessment for included non-randomised studies.

Overall, most studies were judged to have low-to-moderate risk of bias across individual domains, but important concerns remained in several observational cohorts. Moderate risk was most frequent for confounding, participant selection, missing data, and selective reporting. Serious or critical overall risk of bias was identified in a minority of studies, principally because of substantial residual confounding, incomplete adjustment for baseline illness severity and transplant-selection factors, or limitations in outcome measurement and reporting. These findings are consistent with the predominantly retrospective and registry-based nature of the evidence and support cautious interpretation of the pooled estimates (Figure 6 and Figure 7).

## 4. Discussion

The present meta-analysis shows a clear and clinically useful signal. Among carefully selected recipients who underwent liver transplantation, one-year survival in ACLF was 78.8% (95% CI: 70.1–85.4), compared with 86.9% (95% CI: 75.3–93.5) in non-ACLF recipients. The direct comparison confirmed lower one-year survival in ACLF recipients (RR: 0.9295; 95% CI: 0.9179–0.9413; *p* < 0.0001). These findings indicate that ACLF continues to matter after transplantation: it does not make transplantation futile, but its systemic burden is not fully neutralised by replacing the liver.

The pooled 78.8% survival estimate should not be interpreted as the expected outcome for all patients presenting with ACLF. It reflects outcomes in a highly selected subgroup who survived the initial deterioration, underwent transplant assessment, met centre-specific eligibility criteria, received a graft, and were managed in transplant-capable settings. Patients with uncontrolled sepsis, irreversible multiorgan failure, prohibitive frailty, or no access to transplantation are not represented by this estimate. The finding therefore supports transplantation in carefully selected ACLF candidates, while arguing against the assumption that all patients with ACLF derive comparable benefit.

These findings sit well with the broader transplant literature. Several cohorts have shown that selected patients with ACLF can achieve good post-transplant outcomes, even when the pre-transplant course is severe [21,31,32,35,39,40,42,46,47]. Finkenstedt et al. reported excellent post-transplant outcomes in ACLF despite high waitlist mortality, which already suggested that the major barrier is often access to transplantation rather than lack of post-transplant benefit [39]. Sundaram et al., using a large registry-based analysis, similarly showed that patients with severe ACLF may benefit from transplantation, although survival varies according to the number and type of organ failures [46]. More recent data from Tanaka et al. have been more cautious, showing reduced short-term survival after liver transplantation in patients with ACLF when using OPTN data [23]. The present analysis is consistent with both positions. It shows meaningful one-year survival after transplantation, but also confirms that ACLF recipients remain disadvantaged compared with non-ACLF recipients. The message is not “transplant everyone” or “exclude ACLF”. The message is “select better”.

This distinction is important because ACLF is not one disease state. It is a dynamic syndrome. An ACLF patient with controlled infection, improving lactate, reversible renal dysfunction, and limited extrahepatic failure is not the same clinical candidate as a patient with escalating vasopressor requirement, uncontrolled sepsis, mechanical ventilation, and persistent multiorgan failure. The EASL guidelines emphasize this heterogeneity and the need to integrate ACLF grade, organ-failure burden, infection, and clinical trajectory when making transplant decisions [22]. Recent reviews on transplantation for ACLF have also framed the same dilemma: liver transplantation is the only curative treatment, but the decision is constrained by urgency, donor scarcity, perioperative risk, and potential futility [47,48,49,50,51]. The present meta-analysis adds a quantitative estimate to this debate. One-year survival is high enough to justify transplantation in selected ACLF patients, but lower enough than non-ACLF survival to require careful risk stratification.

The high heterogeneity observed in the pooled survival analyses is not a statistical nuisance alone; it is part of the clinical story. Studies differed in ACLF definition, ACLF grade, donor type, regional allocation systems, living versus deceased donor transplantation, and the intensity of pre-transplant critical illness. Living donor cohorts from Asia may represent highly selected candidates with different timing and donor availability than deceased donor registry cohorts from Europe or North America [35,43,47]. Large registry analyses, such as Sundaram et al., carry statistical weight but may include less granular clinical detail regarding infection control, frailty, sarcopenia, vasopressor exposure, and reversibility of organ failure [46]. Smaller single-centre cohorts may provide richer clinical selection detail, but at the cost of limited precision and generalizability [31,32,39,40]. This explains why proportional survival estimates varied widely, while the comparative risk ratio showed only moderate heterogeneity. Within the same transplant environment, ACLF generally remains worse than non-ACLF.

The result also reinforces the limitations of MELD-based allocation when applied to ACLF. MELD captures bilirubin, INR, creatinine, and sodium in its modified versions, but it does not fully capture systemic inflammation, mechanical ventilation, vasopressor dependence, infection severity, sarcopenia, frailty, or dynamic deterioration [52,53,54,55,56,57]. This is why CLIF-SOFA and CLIF-C ACLF have become clinically relevant. They are not perfect, but they move the conversation from “how abnormal are the liver tests?” to “how many organs are failing, and is this trajectory reversible?” [2,4,6,8,17]. The CLIF-C ACLF score is particularly relevant when discussing the edge of futility. Engelmann et al. showed that a CLIF-C ACLF score ≥70 after 48 h of intensive care identified patients with 100% 28-day mortality in their cohort, raising the possibility that this threshold may help define when ongoing intensive support, especially in non-transplant candidates, becomes futile [58]. This should not be used crudely as an automatic exclusion rule. It should, however, make transplant teams pause. A patient beyond the threshold of reversibility may need a different conversation.

The comparison with non-transplanted ACLF is also clinically relevant, even if this meta-analysis focused on transplanted ACLF versus transplanted non-ACLF recipients. Critically ill cirrhotic patients who do not undergo transplantation have very high mortality, particularly when ACLF is complicated by renal failure, infection, sepsis, or circulatory failure [7,9,19,48]. Bernal et al. recently described survival with and without transplantation in critically ill patients with cirrhosis across a twenty-year experience, reinforcing that transplantation can provide survival benefit in selected critically ill patients but that timing and selection are decisive [48]. This fits the practical reality in transplant hepatology: the sickest patient is not always the best candidate, and the best candidate is not always the least sick. The correct candidate is the one in whom transplantation changes the trajectory.

Artificial intelligence and machine learning may become useful in this space because the decision is too complex for linear scores alone. Liver transplantation outcomes depend on recipient factors, donor factors, operative variables, cardiovascular risk, infection, renal dysfunction, frailty, and centre-level practice. A systematic review of machine learning models for liver transplantation showed that these models often achieved satisfactory to excellent discrimination and frequently outperformed traditional scoring systems for outcomes such as mortality, sepsis, acute kidney injury, and graft-related complications [52]. This does not mean that machine learning should replace clinical judgment. It means that the data structure of transplantation is probably better suited to models capable of handling non-linear interactions. ACLF is exactly the type of setting where that matters.

There is already early supporting work in liver disease and transplantation. Machine learning models have been used to predict rebleeding and mortality after oesophageal variceal bleeding, with high reported discrimination in historical cohorts [53]. A later prospective validation study using random forests for one-year mortality after acute oesophageal variceal bleeding showed robust performance, with an AUC of 0.88 in the prospective cohort and good calibration [55]. In transplantation specifically, a random forest model for 30- and 365-day post-liver transplantation mortality reported AUROCs of 0.98 and 0.97, respectively, suggesting that multidimensional pre-transplant and donor data may predict post-transplant mortality better than conventional approaches [54]. Another machine learning model using XGBoost predicted major adverse cardiovascular events after liver transplantation with an AUROC of 0.89 and good calibration, incorporating both hepatic and cardiovascular variables [56]. These studies are not definitive for ACLF transplantation, but they point in the right direction. Future allocation models should probably not rely on MELD, CLIF-C ACLF, donor risk, and frailty as separate silos. They should integrate them.

Sarcopenia should not be considered peripheral to ACLF assessment. Although ACLF is commonly framed through organ failure, infection, and systemic inflammation, skeletal muscle mass and function are also important components of prognosis in cirrhosis and transplantation. Sarcopenia is common among patients listed for liver transplantation and has been associated with poorer survival, functional impairment, malnutrition, and reduced physiological reserve [59,60].

This is particularly relevant in ACLF, where the acute insult occurs in patients whose baseline reserve may already be limited. Two patients with similar MELD or CLIF-C ACLF scores may have very different capacities to tolerate intensive care, surgery, infection, and rehabilitation depending on muscle mass and functional status. A recent systematic review of resistance training in pre- and post-liver transplant patients reported improvements in peak VO_2_, six-minute walk distance, quadriceps muscle thickness, grip strength, fatigue, respiratory pressures, and length of hospital stay in selected studies [59].

Prehabilitation is not always feasible in ACLF because the clinical timeline is often short. However, sarcopenia assessment and early rehabilitation planning should be incorporated into transplant candidacy assessment, postoperative recovery, and longer-term survivorship planning.

The palliative dimension is also important and is frequently addressed too late. In ACLF, palliative care should not be introduced only once transplantation is no longer possible. These patients often experience high symptom burden, recurrent hospitalisations, uncertain trajectories, and substantial family distress. A systematic review of palliative care in end-stage liver disease and ACLF found that early integration may reduce symptom burden, depressive symptoms, readmissions, and length of stay, yet palliative care remains underused and is often delayed until the final weeks of life [57].

This can be particularly difficult in transplant settings because patients and families may interpret palliative care as abandonment, while clinicians may avoid the discussion while there remains a potential opportunity for transplantation. However, these approaches should not be viewed as mutually exclusive. Palliative care can run in parallel with transplant assessment when prognosis is uncertain, especially in ACLF-3, persistent multiorgan failure, or when CLIF-C ACLF approaches thresholds associated with futility [57,58]. A patient may undergo transplant assessment while also receiving active symptom control, family support, and honest prognostic communication.

The clinical implication of this review is therefore not simply that ACLF recipients have lower one-year survival than non-ACLF recipients after transplantation. Rather, post-transplant survival in ACLF remains sufficiently favourable to justify transplantation in carefully selected patients, but sufficiently variable to require more refined selection. Static baseline classification alone is unlikely to be adequate.

Dynamic factors may be more informative, including the trajectory over 24–72 h, response to infection treatment, lactate clearance, renal recovery or dependence, vasopressor dose, ventilatory status, frailty, sarcopenia, and donor quality. This also helps explain why studies using dynamic ACLF assessment have reported useful prognostic information after transplantation [36]. Selection should therefore be guided by reversibility and expected post-transplant survival, rather than urgency alone. This broader approach is biologically justified. ACLF is a systemic inflammatory and multiorgan-failure syndrome, not simply worsening hepatic dysfunction. Oxidative stress may also contribute to this burden, as malondialdehyde has been associated with hepatic encephalopathy severity in decompensated cirrhosis, even without clear discrimination of Child–Pugh class or ascites severity [61]. These observations reinforce the need to assess neurological dysfunction, systemic inflammation, and organ-failure trajectory alongside conventional liver scores when considering transplantation [62,63].

This review has several limitations. The evidence base consisted predominantly of observational cohort and registry studies and is therefore vulnerable to confounding by indication, differences in transplant eligibility, donor selection, centre-level practice, and unmeasured severity of extrahepatic organ failure. ACLF definitions, grade distribution, transplant pathway, donor type, and regional allocation practice varied substantially across studies, contributing to the very high heterogeneity observed in the pooled proportional analyses. We explored the availability of data for subgroup analyses according to ACLF definition, grade, donor type, living versus deceased donor transplantation, and geographic region; however, these variables were inconsistently reported, were often unavailable for the ACLF subgroup, or could not be harmonised across studies. Mechanical ventilation, vasopressor requirement, renal replacement therapy, frailty, sarcopenia, and donor characteristics were similarly reported too inconsistently to support reliable subgroup analyses or meta-regression. The pooled one-year survival estimate should therefore be interpreted as a broad summary across heterogeneous transplant settings rather than as an expected outcome for an individual patient. Importantly, transplanted ACLF recipients represent a highly selected subgroup of the wider ACLF population: they survived the initial deterioration, underwent transplant assessment, met centre-specific eligibility criteria, and received an organ. These findings should not be extrapolated to unselected ACLF populations or to patients with uncontrolled sepsis, irreversible multiorgan failure, or no access to transplantation. The literature search was conducted in PubMed, SciELO, and the Cochrane Library, supplemented by reference-list screening; broader database coverage might have identified additional studies. In addition, some studies did not provide extractable paired ACLF and non-ACLF one-year survival data and were retained only for qualitative synthesis. Finally, although funnel plot inspection and Egger’s test did not suggest relevant small-study effects, these methods have limited power in a small and clinically diverse evidence base.

## 5. Conclusions

This meta-analysis shows that ACLF still matters after liver transplantation. Among carefully selected transplant recipients, one-year survival was clinically meaningful, supporting transplantation as a valid and often life-saving option. However, survival remained lower than in non-ACLF recipients, indicating that the systemic burden of ACLF is not fully corrected by replacing the liver. These findings should not be interpreted as evidence that all patients with ACLF will derive comparable benefit from transplantation; rather, they apply to the selected subgroup who reached transplant assessment, met eligibility criteria, and received a graft.

ACLF should therefore not be treated as an automatic exclusion criterion, but neither should it be considered a uniform indication for transplantation. Selection must be dynamic and should integrate ACLF grade, organ-failure burden, infection control, renal replacement therapy, vasopressor and ventilatory support, frailty, sarcopenia, donor quality, and clinical trajectory over time. Future prospective studies and transplant registries should report these factors more consistently to identify which patients benefit most, when transplantation should occur, and when the window for meaningful benefit has closed. Allocation models should move beyond static liver scores by incorporating urgency, reversibility, expected benefit, functional reserve, and post-transplant recovery. Artificial intelligence-supported models may further assist by integrating complex, non-linear recipient, donor, and trajectory data to improve prognostication and support more individualized allocation decisions, although they will require rigorous prospective validation before clinical implementation. ACLF is not the end of the transplant conversation; it is where the conversation must become more precise.

## Figures and Tables

**Table 1:** Characteristics of included studies and extracted one-year post-transplant survival data.

Study	Country of Origin	One-Year Survival After LT in ACLF Patients	Clinical Scenario	One-Year Survival After LT in Non-ACLF Patients
Cronst et al., 2023 [31]	Brazil	20	LT for ACLF versus other indications	296
Goosmann et al., 2020 [32]	Germany	50	Post-LT survival in ACLF	48
Chun Xia-Zhu et al., 2022 [33]	China	170	One-year follow-up after LT	NA
Baptiste-Michard et al., 2017 [34]	Germany	36	Post-transplant mortality/survival	23
Moon et al., 2017 [35]	Korea	145	MELD score and adult living donor LT outcomes	123
Zhang et al., 2023 [36]	China	137	Dynamic ACLF assessment and one-year post-LT outcome	454
O’Leary et al., 2024 [37]	USA	246	LT outcomes in ACLF	NA
Alshuwaykh et al., 2020 [38]	USA	20	Urgent LT evaluation and outcomes	NA
Finkenstedt et al., 2013 [39]	Austria	28	Post-transplant outcomes in ACLF	331
Iacob et al., 2020 [40]	Romania	57	Pre- and post-LT outcomes in ACLF	158
Laique et al., 2020/2021 [41]	USA	NA	LT access and outcomes in ACLF	NA
Bahirwani et al., 2011 [42]	USA	117	ACLF before LT and post-transplant outcomes	146
Vu et al., 2024 [43]	Vietnam	42	Living donor LT for ACLF	NA
Yun et al., 2023 [44]	Korea	NA	Living donor and deceased donor LT	NA
Kwon et al., 2022 [45]	Korea	709	Critically ill LT patients with ACLF-related analysis	NA
Sundaram et al., 2019 [46]	USA	18,291	Registry-based LT outcomes	27,085
Chok et al., 2017 [47]	China	597	Living donor LT	636

NA = not available.

## Data Availability

No new data were created or analyzed in this study. Data sharing is not applicable to this article.

## Supplementary Materials

The following supporting information can be downloaded at: https://www.mdpi.com/article/10.3390/diagnostics16142206/s1. Table S1: PRIMSA 2020 Checklist.
